# Correlation analysis of pituitary sagittal height, anterior posterior diameter, coronal width, and pituitary stalk abnormalities with dwarfism based on MRI measurement

**DOI:** 10.1515/med-2026-1448

**Published:** 2026-06-11

**Authors:** Bing Zhu, Danqiong Deng, Yong Wei, Feng Li, Jingbo Deng, Zidong Lv, Xiaohua Fu

**Affiliations:** Radiology Department, Hainan Women and Children’s Medical Center, Haikou, China; Department of Radiology, Hainan Modern Women and Children’s Hospital, Haikou, China; Department of Radiology, Central South University Xiangya School of Medicine Affiliated Haikou Hospital, Haikou, China

**Keywords:** short stature, pituitary, pituitary stalk, magnetic resonance imaging, data correlation

## Abstract

**Objectives:**

Based on MRI examination, to explore the relationship between pituitary sagittal height (PSH), anterior posterior diameter (APD), coronal width (CW), and pituitary stalk abnormalities (PSA) and short stature.

**Methods:**

A retrospective study was performed on 257 children with suspected short stature admitted to hospitals from January 2020 to January 2024. Clinical data of the patient were collected, and MRI was used to measure the patient’s PSH, APD, CW, and PSA. Spearman correlation analysis and ROC curve analysis were used to verify the relationship between PSH, APD, CW, and PSA, and short stature, as well as their predictive performance for short stature. Positive cases are defined as children diagnosed with short stature who appear 2 standard deviations (<−2SD) or less below average height for their age and sex in height measurements accompanied by abnormal growth hormone levels. In contrast, negative cases are those children whose height is within the normal range, that is, height not less than 2 standard deviations (>−2SD) below the average height for their age and sex, and whose growth hormone levels are within the normal range.

**Results:**

The GH peak values of<−2SD group, −2SD–3SD group, and >−3SD group, were 7.16 (4.58, 10.03) ng mL^−1^, 6.75 (4.60, 8.87) ng mL^−1^, and 6.54 (4.08, 9.27) ng mL^−1^, respectively, showing a gradually decreasing trend, but the overall difference has no meaning (p>0.05). There was p<0.05 in PSH and pituitary volume among the three groups of patients, while there was p>0.05 in pituitary APD, CW, and PSA. The PSH and pituitary volume in the <−2SD group obviously surpassed −2SD∼−3SD groups (p<0.05). PSH was inactively related to short stature (*ρ*=−0.203, p=0.001), but not significantly correlated with pituitary APD, CW, and PSA (p>0.05). Pituitary volume has negative connection with short stature (*ρ*=−0.216, p<0.001). The AUC of PSH and combined diagnosis for predicting short stature were both greater than 0.6 and less than 0.7. PSH was positively correlated with IGF-1 (r=0.381, p<0.001) and TSH (r=0.377, p<0.001).

**Conclusions:**

Pituitary APD, CW, and PSA are not associated with short stature, while PSH is associated with short stature. There is an obvious difference in PSH between children with non-short stature and those with short stature, but the role of PSH in predicting short stature and distinguishing short stature types is relatively poor.

## Introduction

Short stature is a group of growth and development disorders caused by genetic or disease factors, characterized by short stature and slow growth in children. Internationally, it is widely believed that if a child’s length/height is less than 2 standard deviations (SD) from the median of the World Health Organization’s child growth standards, it is considered short stature. According to statistics, the Chinese age standardized prevalence and occurrence rate of short stature are 3.70 and 2.69 %. Among them, the prevalence of short stature in developed, moderately developed, and underdeveloped areas is 2.60 , 3.72, and 4.69 %. The prevalence of short stature in urban and rural areas is 2.23 and 5.12 % [1]. Although the incidence and prevalence rate of short stature in China are lower than the world average [2], short stature has a serious effect on child’s health. For example, short stature can lead to psychological disorders such as inferiority complex and social difficulties in children [3], and can also increase the risk of premature birth in female children as adults [4]. Therefore, early identification of short stature during childhood is vital in reducing the occurrence of physical and mental illnesses in children, and should become an essential component of child’s health plans.

The pathogenesis of short stature is complex and diverse. Taking effective measures to clarify the pathogenesis is crucial for diagnosing, differentiating, and treating short stature. The pituitary gland (PG) is closely related to growth and development, and pituitary lesions can lead to abnormal secretion of growth hormone [5], 6], which in turn can cause short stature. Numerous reports both in China and foreign countries indicate that the PG can serve as an important indicator for the clinical diagnosis of short stature. Research has found that pituitary lesions in children with short stature are characterized by pituitary MRI features like anterior pituitary hypoplasia, Rathke’s cleft cyst, and vacuolar sella turcica [7]. Shu et al. constructed a predictive model using pituitary MRI radiomics, which performed well in predicting short stature growth hormone deficiency [8]. Clinical trials have also shown that after treatment with growth hormone, the volume of the PG, basal ganglia, and marginal structures in children with isolated growth hormone deficiency gradually recovers to the size of healthy children [9]. Kessler et al.’s study showed that pituitary volume increases with age in children, but compared to healthy children, children with growth hormone deficiency and idiopathic short stature have a smaller increase in pituitary volume [10]. However, there are currently few reports indicating the relationship between pituitary sagittal height (PSH), anterior posterior diameter (APD), coronal width (CW), and short stature. Additionally, many studies have confirmed that developmental delay in children is associated with the occurrence of pituitary stalk interruption syndrome [11], 12], but whether it can be used as a predictive indicator for short stature is not yet clear.

To further clarify the value of PG in the diagnosis of short stature, this study explores the relationship between PSH, APD, CW, and pituitary stalk abnormalities (PSA) and short stature based on MRI measurements, to provide new references for early identification of short stature.

## Materials and methods

### Sample size estimation

The sample size was estimated using the “Spearman’s Rank Correlation Tests” module in PASS 15.0 software, and the testing level was *α*=0.05 and testing performance was *β*=0.8. In the preliminary experiment, the correlation coefficient between PSH and short stature was *α*=0.3, and the calculated sample size was n≥166 cases. In addition, considering the missing sample data, an additional 20 % of the data was included, resulting in a final sample size of n≥208 cases.

### Case information

**Figure 1:**
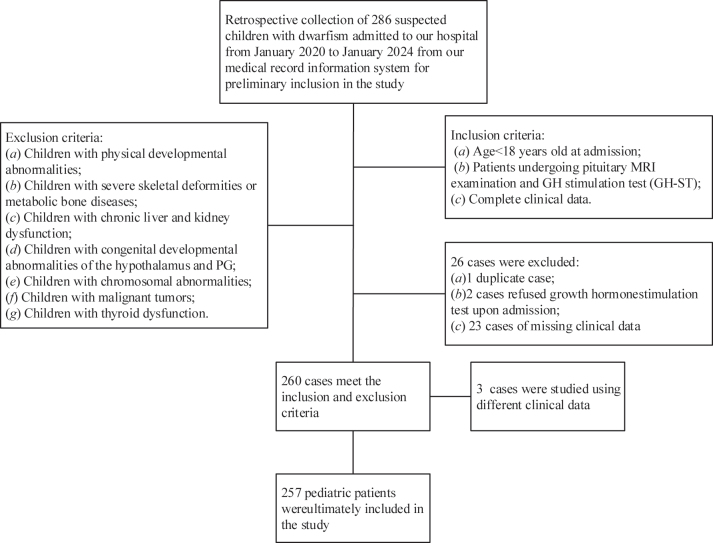
Sample inclusion process diagram.

Based on the sample size estimation method, 286 suspected cases of short stature admitted from Jan. 2020 to Jan. 2024 were retrospectively collected from the hospital’s medical record information system for preliminary inclusion in the study. Inclusion criteria: (*a*) Age <18 years old at admission; (*b*) Patients undergoing pituitary MRI examination and GH stimulation test (GH-ST); (*c*) Complete clinical data. Exclusion criteria: (*a*) Children with physical developmental abnormalities; (*b*) Children with severe skeletal deformities or metabolic bone diseases; (*c*) Children with chronic liver and kidney dysfunction; (*d*) Children with congenital developmental abnormalities of the hypothalamus and PG; (*e*) Children with chromosomal abnormalities; (*f*) Children with malignant tumors; (*g*) Children with thyroid dysfunction. According to the above criteria, 1 duplicate case was excluded, 2 cases refused to undergo GH-ST upon initial admission, and 23 cases had missing clinical data. A total of 257 samples were ultimately included. The sample inclusion process is detailed in Figure 1.

### Investigation methods

The experiment collected clinical data of the children, including gender, age (including months, expressed as decimals in this study, such as 7 years and 3 months as 7.25 years), bone age (expressed as same as the age), height, BMI, father height, mother height, target height, insulin-like growth factor 1 (IGF-1), 25 hydroxyvitamin D [25(OH)D], thyroid stimulating hormone (TSH), 17α-hydroxyprogesterone (17α-OHP)), mother’s pregnancy and delivery frequency (expressed as G and P respectively, such as G1P1 indicating pregnancy and delivery once), whether the child was born full-term (pregnancy time 37–42 weeks), history of hypoxia and asphyxia at birth, method of delivery, body length at birth, BMI at birth, GH-ST results (0 min, 30 min, 60 min, 90 min, and 120 min after administration), PSH, APD, CW, PSA and whether accompanied by empty sella syndrome.

### Serum index detection method

In the early morning, 5 mL of fasting elbow vein blood was taken from the patient, centrifuged at 4 °C and 3,000 rpm for 10 min, and the upper layer of serum was saved for testing. (*a*) The detection methods for IGF-1 and 17 α-OHP were performed using the Infinity LC Clinical Edition liquid chromatography tandem mass spectrometry system to detect IGF-1 (reference values: 55–539 ng mL^−1^ for males and 55–567 ng mL^−1^ for females) and 17 α-OHP (0.2–2.7 ng mL^−1^). (*b*) The detection methods for 25(OH)D and TSH were carried out using a Cobas 8000 e 602 fully automatic chemiluminescence immunoassay analyzer to detect 25(OH)D (reference value: 30–200 nmol L^−1^) and TSH (reference value: 0.49–4.67 mIU L^−1^).

### GH stimulation test

The patient was fasted overnight after 20:00 the day before the experiment, and 3 mL of fasting elbow vein blood was drawn from the patient while lying in bed the next morning (0 min after administration). Subsequently, 0.5 g/kg 25 % arginine hydrochloride (20 mL: 5 g, National Medical Products Administration Approval H42023001, Wuhan Fuxing Biopharmaceutical Co., Ltd.) was diluted with 0.9 % saline to a total volume of 200 mL. Intravenous infusion was used and completed within 30 min. Calculate the time from the start of infusion, and draw 3 mL of fasting elbow vein blood from the other upper limb at 30, 60, 90, and 120 min after administration. Serum was obtained using the same method as described above, and GH was detected using a Cobas 8000 e 602 fully automated chemiluminescence immunoassay analyzer (neonatal period: 5–40 ng mL^−1^, infant period: 10–50 ng mL^−1^, preschool period: 1–20 ng mL^−1^, pre-school period: 0.2–10 ng mL^−1^, prepubertal period: 0.2–20 ng mL^−1^).

### Other pituitary hormone detection methods and stimulation experiments

Thyroid stimulating hormone (TSH) test method: This method uses specific antibodies bound to markers to measure serum TSH concentration. After the sample reacts with the immobilized anti-TSH antibody, the binding complex is washed to remove the unbound substance, followed by the addition of a luminescent substrate, the release of photons by enzymatic reaction, and the light signal is measured by photometer to determine the TSH concentration.

Methods for detecting Luteinizing Hormone (LH) and Follicle Stimulating Hormone (FSH): The secretion response of LH and FSH was evaluated by intravenous or subcutaneous injection of GnRH (100 μg). Blood samples were taken at baseline (before injection) and 30 and 60 min after injection to measure changes in LH and FSH levels.

Adrenocorticotropin (ACTH) detection method: The response of ACTH is observed by intravenous injection of CRH (usually 100 μg). Blood samples were collected before, 30 and 60 min after injection to measure changes in ACTH.

### Pituitary measurement methods

**Figure 2:**
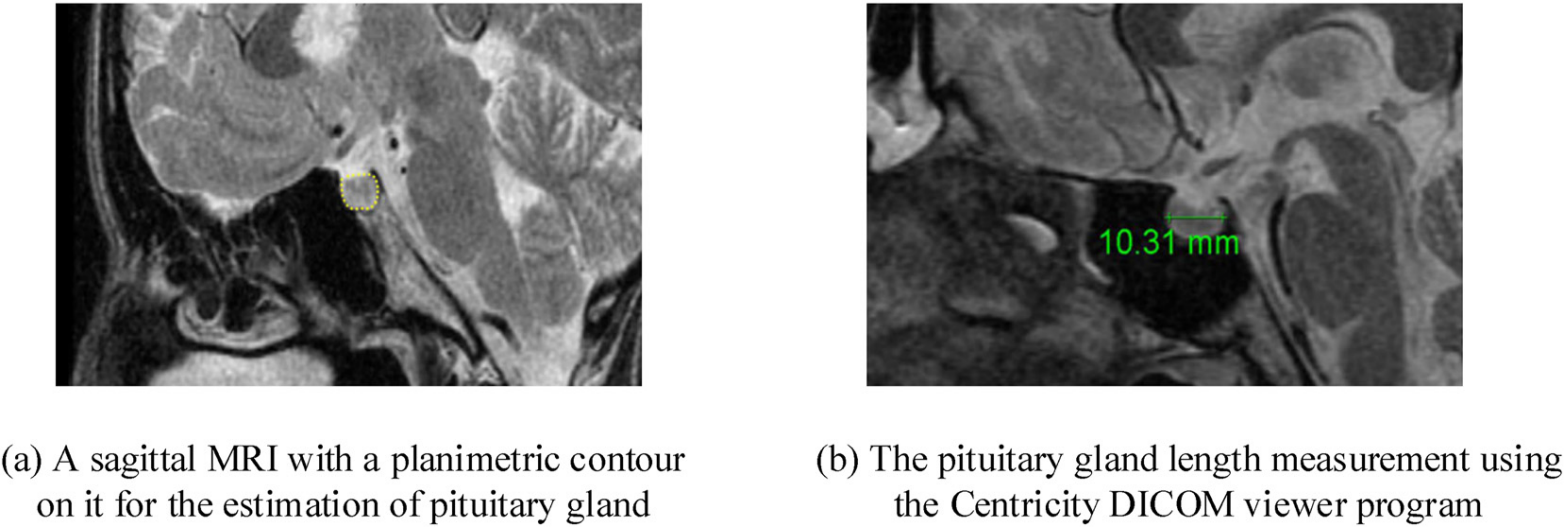
Pituitary estimation diagram.

MRI examination was used to measure PSH, APD, CW, and PSA. MRI can detect developmental conditions, structural abnormalities, and lesions (such as tumors, cysts, or dysplasia) of the pituitary gland that may result in abnormal production of growth control hormones, which can affect the growth and development of children. In addition, MRI is radiation-free and has high resolution, which can clearly show the fine structure of the pituitary gland, ensuring the accuracy of the evaluation. By combining imaging findings with clinical manifestations, MRI can provide a more comprehensive diagnostic basis in the assessment of dwarfism, help identify the underlying etiology of growth disorders, and guide subsequent management and treatment measures more effectively. The MRI results in the study were designed to provide patients with short stature with more detailed information on the structure of the pituitary gland, thereby assisting physicians in assessing the potential cause of growth hormone deficiency (GHD). Despite the higher cost of MRI, its high resolution and radiation-free nature make it particularly suitable for children who require long-term monitoring, especially when there is uncertainty about other clinical evaluations. Ultimately, MRI should be used as part of a comprehensive assessment tool, combined with clinical manifestations and biochemical tests, to guide more personalized treatment and management. The Ingenia Elition X medical MRI system (National Medical Equipment Injection 20203060201, Philips, Netherlands) was utilized to perform pituitary coronal and sagittal SE sequence T_1_WI and T_2_WI plain scans. Scanning parameters: TR 500 ms, TE 10 ms, Layer spacing 0.3 mm, layer thickness 3 mm, excitation frequency 3, field of view with 180 × 180 mm, contrast agent 0.1 mol/kg Gd DTPA. The experiment used a double-blind method for film reading, in which two experienced radiologists from the hospital were selected to independently measure PSH, APD, and CW without knowing the patient’s identity and condition. The mean of the two measurement results was taken as the final result, and the morphology of the PG was also observed. If there was a disagreement on the observation results, it needed to be determined through consultation between two radiologists. According to PSH, APD, and CW, the volume of the PG was calculated. Pituitary volume=(Height × Length × Width)/2 [13]. The specific estimation of pituitary gland is shown in Figure 2.

### Diagnosis criteria for short stature

Short stature is a group of growth and development disorders due to genetic or pathological factors, characterized by significantly short stature in children. According to World Health Organization (WHO) criteria, short stature is generally defined as a child’s height being 2 SD below the average height of children of the same age and sex. Short stature can occur for a variety of reasons, including growth hormone deficiency, abnormal bone development, genetic disorders (such as Marfan syndrome or acromegaly), and endocrine disorders.

Referring to the diagnostic recommendations for short stature provided by the Growth Hormone Research Society (GRS) [14], combined with clinical experience in diagnosing short stature in China, children who meet the following criteria are diagnosed with short stature: (*a*) short stature, with a height below 2 SD of the mean height of child of the similar age and gender; (*b*) Slow growth, height growth rate <5 cm/year; (*c*) Bone age is 2 years or more behind actual age; (*d*) Abnormal results in the detection of growth related serum markers, such as IGF-1 and GH levels below normal. This study divided the children into three groups based on the SD fold difference in their average height, namely the <−2SD group, −2SD∼−3SD group, and >−3SD group. The rationale for dividing children into <−2SD, −2SD to −3SD, and >−3SD groups is the quantification of height differences into SD multiples according to the WHO child growth standards in order to more precisely assess children’s height relative to normal children of the same age. This grouping approach can help identify different levels of nanosomia, from mild to severe, and thus assess the potential effects of GHD and the corresponding clinical manifestations. At the same time, by dividing different standard deviation groups, different levels of growth and development problems can be revealed, so that clinicians can develop more personalized assessment and intervention programs according to the characteristics of each group.

According to the results, the pituitary gland is divided into three conditions: “abnormal”, “no abnormal” and “other abnormal”. “Abnormal” means that the patient is found to have a definite pituitary lesion on imaging, usually affecting the structure and function of the pituitary gland. These include Rathke’s cyst, empty saddle syndrome, and pituitary dysplasia. “No abnormality” means that after imaging examination, no changes or lesions in the pituitary structure are found, and the shape and position of the pituitary gland are normal. The term “other abnormalities” refers to imaging findings that are uncommon or difficult to categorize clearly and that do not meet the conventional definition of “abnormal” or “abnormal.” Including pituitary tract abnormalities, calcification and suspicious imaging.

### Standard deviation analysis method of IGF-1 data

Descriptive statistical method was used to analyze the standard deviation of IGF-1 data. First, IGF-1 measurements were collected for all children and their mean and standard deviation (SD) were calculated. The calculated standard deviations were then compared with specific clinical reference values for IGF-1 to assess whether IGF-1 levels were normal during growth and development in children and to further analyze its association with short stature. In this way, a clearer picture of the fluctuating range of IGF-1 levels can be obtained to support a comprehensive assessment of children with growth hormone deficiency.

In order to consider the effects of age and sex on IGF-1 levels, IGF-1 measurements of all children were stratified by sex and age according to established clinical reference values. These reference values are developed according to the growth and development characteristics corresponding to different ages and genders, and are used as benchmarks for comparison in data analysis. The study obtained the corresponding normal range for each age group and gender group, and calculated the deviation of each child’s IGF-1 values from these reference values to determine their standard deviation score (Z-score), thereby identifying whether IGF-1 levels were abnormal. This approach ensures that the assessment of IGF-1 levels is more in line with the physiological norm, thereby improving the accuracy of determining the effects of growth hormone deficiency.

### Statistics

The data processing and analysis used SPSS 27.0in the experiment. Kolmogorov-Smirnov test was utilized to verify whether the quantitative data accord to a normal distribution, which wasdisplayed by mean±standard deviation ( $\overset{®}{x}\pm s$ ). Perform *t*-test between 2 groups and one-way ANOVA test between multiple groups. The skewed distribution data weredenoted by median and quartiles [*M*(*Q*_1_, *Q*_3_)]. Perform Mann Whitney U test in both groups. Perform Kruskal-Wallis test among multi-groups. The count data were expressed in terms of cases and percentage [n (%)], and a *χ* ^2^-test was performed. Wilcoxon rank sum test was performed on grade data. Spearman (*ρ* value) or Pearson (*r* value) was adopted for correlation analysis. GraphPad Prism 9 was taken to draw scatter plots and correlation curves. p<0.05 means a statistically obvious difference.

The experimental protocol was established, according to the ethical guidelines of the Helsinki Declaration and was approved by the Hainan Women and Children’s Medical Center. HNWCMC L.S. [2025] No. [119]. Informed consent was obtained from all individuals included in this study, or their legal guardians or wards.

### Research ethics

The experimental protocol was established, according to the ethical guidelines of the Helsinki Declaration and was approved by the Hainan Women and Children’s Medical Center. HNWCMC L.S. [2025] No. [119].

### Informed consent

Informed consent was obtained from all individuals included in this study, or their legal guardians or wards.

## Results

### Clinical data among three groups of pediatric patients

In the overall comparison, there were significant differences among the three groups of children in terms of age, height, BMI, 25(OH)D, and mode of delivery (p<0.05). Inter-group comparisons showed that children in the −2SD to −3SD group had lower age, 17α-OHP, height, BMI, and bone age compared to the <−2SD group, while the differences in 25(OH)D, age, and bone age were greater in the <−2SD group, and these differences were statistically significant (p<0.05). Children in the >−3SD group had lower height, BMI, and paternal height compared to the <−2SD group, with statistically significant differences (p<0.05). Please refer to Table 1 for details.

**Table 1:** Clinical data of children with height SD differences of different multiples [n(%), M (Q_1_, Q_3_), ( $\overset{®}{x}\pm s$ )].

Item	<−2SD group (n=61)	−2SD∼−3SD group (n=153)	>−3SD group (n=43)	χ^2^/F/H	p-Value
Gender				1.711	0.425
Male	30 (49.18)	90 (58.82)	25 (58.14)		
Female	31 (50.82)	63 (41.18)	18 (41.86)		
Age/year	8.92 (5.18, 7.11)	7.17 (5.42, 9.52)^b^	9.08 (5.63, 10.69)	9.238	0.010
Bone age/year	8.02 ± 2.64	6.61 ± 2.56^b^	7.26 ± 2.55	4.725	0.010
Age and bone age difference/year	0.65 ± 1.40	1.22 ± 1.08^b^	1.30 ± 1.22^b^	4.287	0.015
Height/cm	125.90 (114.73, 131.73)	114.00 (103.00, 124.48)^b^	116.00 (103.50, 123.98)^b^	22.691	<0.001
BMI/kg	23.00 (19.60, 27.78)	18.20 (15.50, 22.78)^b^	20.20 (15.25, 22.88)^b^	24.279	<0.001
Father’s height/cm	169.00 (165.00, 172.00)	168.00 (163.75, 170.00)	165.00 (163.00, 168.00)^b^	5.803	0.055
Mother’s height/cm	157.00 (153.00, 160.00)	155.00 (152.00, 158.00)	155.00 (150.00, 159.00)	3.616	0.164
Target height/cm	162.00 (155.88, 168.00)	164.00 (155.00, 168.63)	161.50 (156.13, 167.38)	0.496	0.780
IGF-1^a^/ng mL^−1^	131.00 (88.20, 215.00)	120.50 (80.50, 163.00)	122.00 (73.08, 171.00)	2.445	0.294
25(OH)D^a^/nmol L^−1^	69.05 (57.90, 77.60)	74.30 (64.55, 90.41)^b^	70.55 (60.05, 81.15)	7.488	0.024
TSH^a^/mIU L^−1^	5.48 (3.670, 7.63)	5.94 (4.03, 7.11)	7.58 (4.91, 8.54)	2.138	0.343
17α-OHP^a^/ng mL^−1^	1.33 (0.75, 1.97)	0.89 (0.63, 1.62)^b^	0.83 (0.36, 1.64)	4.158	0.125
Mother pregnant/giving birth				13.896	0.178
G1P1	25 (40.98)	68 (44.44)	16 (37.21)		
G2P1	0 (0.00)	4 (2.61)	4 (9.30)		
G2P2	6 (9.84)	13 (8.50)	4 (9.30)		
G3P2	0 (0.00)	1 (0.65)	0 (0.00)		
G3P3	0 (0.00)	0 (0.00)	1 (2.33)		
Unknown	30 (49.18)	67 (43.79)	18 (41.86)		
Whether it is full-term at birth				2.279	0.685
No	4 (6.56)	4 (2.61)	2 (4.65)		
Yes	36 (59.02)	100 (65.36)	26 (60.47)		
Unknown	21 (34.43)	49 (32.03)	15 (34.88)		
History of hypoxia and asphyxia at birth				5.338	0.254
No	45 (73.77)	125 (81.70)	38 (88.37)		
Yes	0 (0.00)	2 (1.31)	0 (0.00)		
Unknown	16 (26.23)	26 (16.99)	5 (11.63)		
Delivery method				12.138	0.016
Caesarean section	8 (13.11)	13 (8.50)	0 (0.00)		
Natural childbirth	22 (36.07)	83 (54.25)	19 (44.19)		
Unknown	31 (50.82)	57 (37.25)	24 (55.81)		
Body length at birth/cm				7.523	0.111
<50	5 (8.20)	14 (9.15)	3 (6.98)		
≥50	1 (1.64)	20 (13.07)	3 (6.98)		
Unknown	55 (90.16)	119 (77.78)	37 (86.05)		
BMI at birth/g				6.801	0.340
<1,500	0 (0.00)	1 (0.65)	1 (2.33)		
1,500−2,500	5 (8.20)	10 (6.54)	6 (13.95)		
>2,500	27 (44.26)	85 (55.56)	19 (44.19)		
Unknown	29 (47.54)	57 (37.25)	17 (39.53)		
Accompanied by empty sella syndrome	6 (9.84)	7 (4.58)	1 (2.33)	3.321	0.190

^a^indicates the presence of missing data; ^b^refers to the comparison with children in <−2SD, p<0.05.

### GH-ST results among three groups of pediatric patients

**Figure 3:**
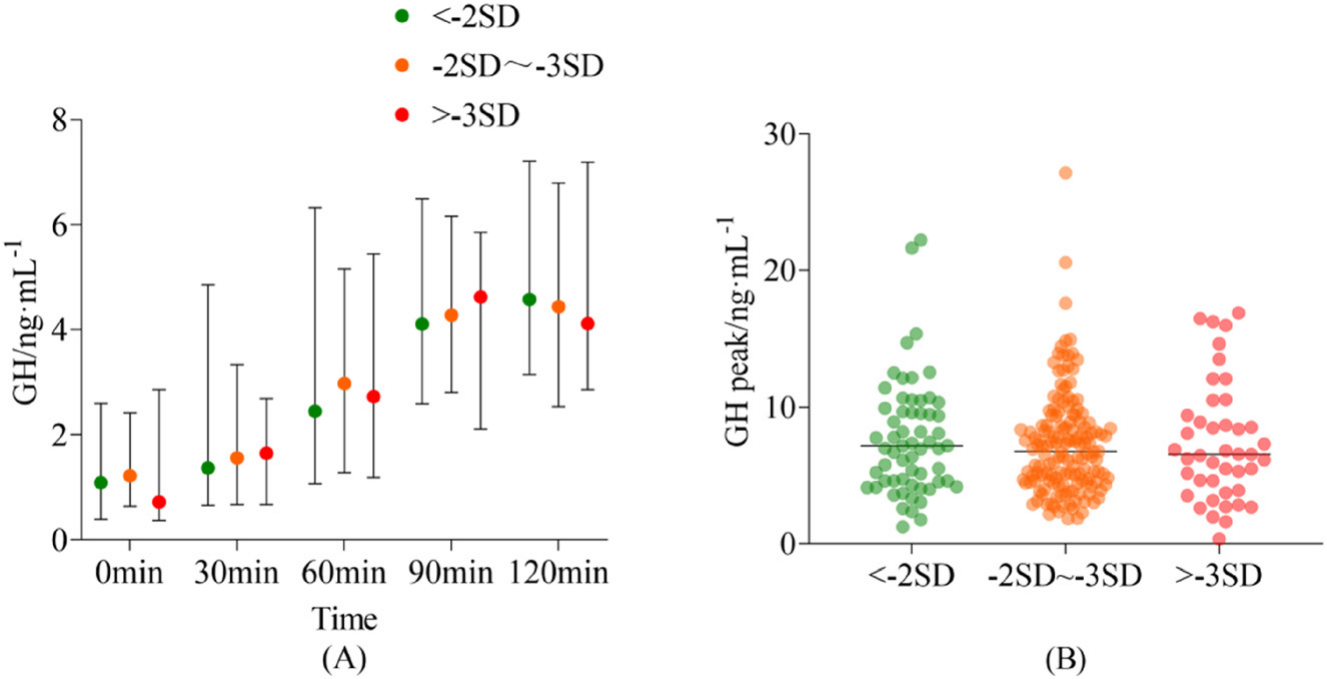
GH-ST results for three groups of children. Note: (A) and (B) are scatter plots of GH levels and GH peak values for three groups at different periods; Green=<−2SD group, Orange=−2SD∼−3SD group, Red=>−3SD group.

Overall, there were no significant differences in GH levels at different time points of GH-ST among the three groups of children (p>0.05), as shown in Figure 3 (A). The GH peak values for the <−2SD group, −2SD to −3SD group, and >−3SD group were 7.16 (4.58, 10.03) ng·mL^−1^, 6.75 (4.60, 8.87) ng·mL^−1^, and 6.54 (4.08, 9.27) ng·mL^−1^, respectively, showing a gradual decreasing trend, but overall no significant difference was observed (p>0.05), as illustrated in Figure 3 (B).

In the comparison of Table 2, there was p>0.05 in the proportion of GH peak among the three groups of GH-STs.

**Table 2:** GH peak proportion in GH-ST among three groups of children [n(%)].

GH peak value	<−2SD group (n=61)	−2SD∼−3SD group (n=153)	>−3SD group (n=43)	χ^2^	p-Value
<5 ng mL^−1^	19 (31.15)	46 (30.07)	13 (30.23)	1.946	0.746
5–10 ng mL^−1^	27 (44.26)	80 (52.29)	20 (46.51)		
>10 ng mL^−1^	15 (24.59)	27 (17.65)	10 (23.26)		

As compared with Table 3, TSH levels showed significant differences among different height standard deviation groups (p<0.05). There were no significant differences in LH and FSH levels among all groups (p>0.05). There was no significant difference in ACTH among the three groups (p>0.05).

**Table 3:** Measurement results of different pituitary hormones in the three groups of children[*M*(*Q*_1_, *Q*_3_)]

Hormone	<−2SD group (n=61)	−2SD∼−3SD group (n=153)	>−3SD group (n=43)	p-Value
TSH, mIU/L	5.48 (3.67, 7.63)	5.94 (4.03, 7.11)	7.58 (4.91, 8.54)	<0.05
LH, mIU/L	0.35 (0.20, 0.60)	0.32 (0.15, 0.55)	0.50 (0.25, 0.75)	0.321
FSH, mIU/L	1.00 (0.50, 2.00)	0.89 (0.40, 1.90)	1.25 (0.60, 2.00)	0.457
ACTH, pg/mL	18.0 (10.0, 25.0)	17.5 (9.0, 24.0)	20.0 (12.0, 28.0)	0.231

### Comparison of pituitary measurement results among three groups of pediatric patients

In the comparison of PSH and pituitary volume, significant differences were observed among the three groups (p<0.05), while no significant differences were found for APD, CW, and PSA (p>0.05). The inter-group comparison results in Table 4 show that the PSH and pituitary volume in the <−2SD group were significantly higher than those in the −2SD to −3SD group, with statistical significance (p<0.05).

**Table 4:** Pituitary measurement results of three groups of children [*M*(*Q*_1_, *Q*_3_), ( $\overset{®}{x}\pm s$ ), n(%)].

Pituitary measurement parameters	<−2SD group (n=61)	−2SD∼−3SD group (n=153)	>−3SD group (n=43)	χ^2^/F/H	p-Value
PSH/mm	4.00 (3.17, 5.12)	3.50 (2.88, 4.11)^a^	3.58 (2.73, 4.65)	12.239	0.002
APD/mm	5.48 ± 1.05	5.42 ± 1.14	5.14 ± 1.44	1.215	0.298
CW/mm	10.34 ± 1.66	9.90 ± 1.79	10.03 ± 1.71	1.357	0.259
Pituitary volume/mm^3^	243.59 ± 107.40	190.58 ± 85.04^a^	216.67 ± 180.07	5.055	0.007
Pituitary abnormality				6.036	0.196
No abnormalities	34 (55.74)	95 (62.09)	20 (46.51)		
Abnormality	1 (1.64)	0 (0.00)	1 (2.33)		
Other anomalies	26 (42.62)	58 (37.91)	22 (51.16)		

^a^means that compared with <−2SD, children, p<0.05.

### Relationship between PSH, APD, CW, PSA and short stature

**Figure 4:**
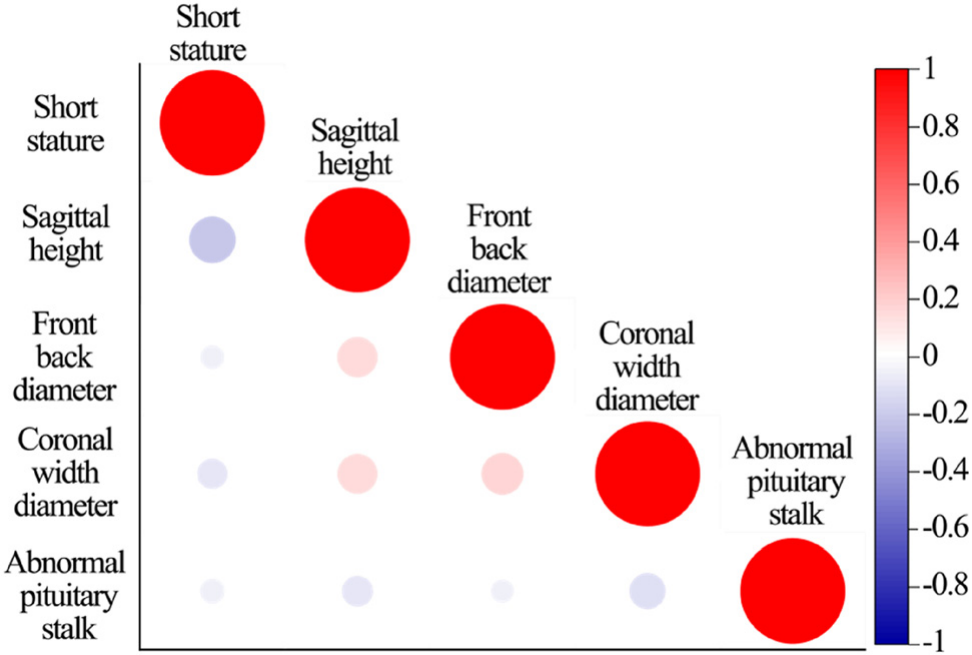
Heat map of the correlation between PSH, APD, CW, PSA and short stature.

In the correlation analysis, PSH showed a negative correlation with short stature (*ρ*=−0.203, p=0.001), while no correlation was found with APD, CW, and PSA (p>0.05), as shown in Figure 4. Pituitary volume also exhibited a negative correlation with short stature (*ρ*=−0.216, p<0.001).

### The predictive performance of PSH, APD, CW, and PSA for short stature

**Figure 5:**
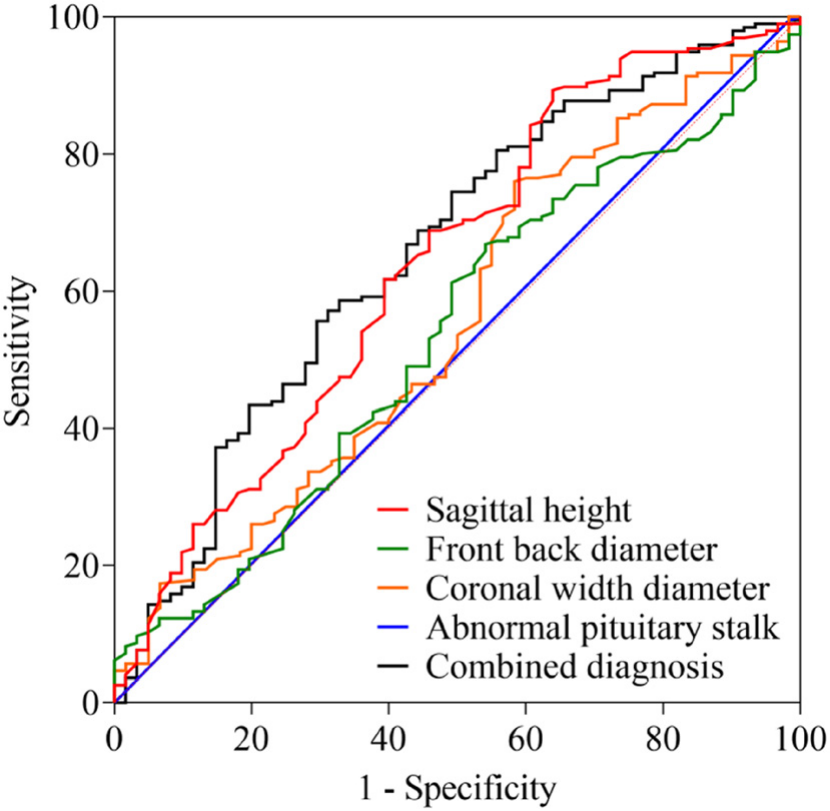
ROC curves for predicting short stature using PSH, APD, CW, and PSA.

In the ROC curve analysis, only the AUC of PSH and joint diagnostic prediction for short stature were >0.6, and both were <0.7, as list in Table 5 and Figure 5. In the study, “combined diagnosis” refers to a comprehensive evaluation method that combines multiple clinical indicators and imaging results to improve the diagnostic accuracy of short stature. This diagnostic method combines PSH, pituitary volume, related hormone levels and clinical manifestations of the patient.

**Table 5:** ROC curve parameters for predicting short stature using PSH, APD, CW, and PSA.

Variables	AUC	SE	p-Value	Youden J	Best truncation value	Sensitivity	Specificity
PSH	0.638	0.042	0.001	0.254	≤4.58 mm	89.29 %	36.07 %
APD	0.535	0.042	0.411	0.127	≤5.82 mm	66.84 %	45.90 %
CW	0.557	0.043	0.184	0.177	≤11.01 mm	76.02 %	41.67 %
PSA	0.506	0.043	0.895	0.011	–	99.49 %	1.64 %
Combined diagnosis	0.658	0.041	<0.001	0.261	>−2.88	55.61 %	70.49 %

### The relationship between PSH, APD, CW, PSA and short stature related serum markers

**Figure 6:**
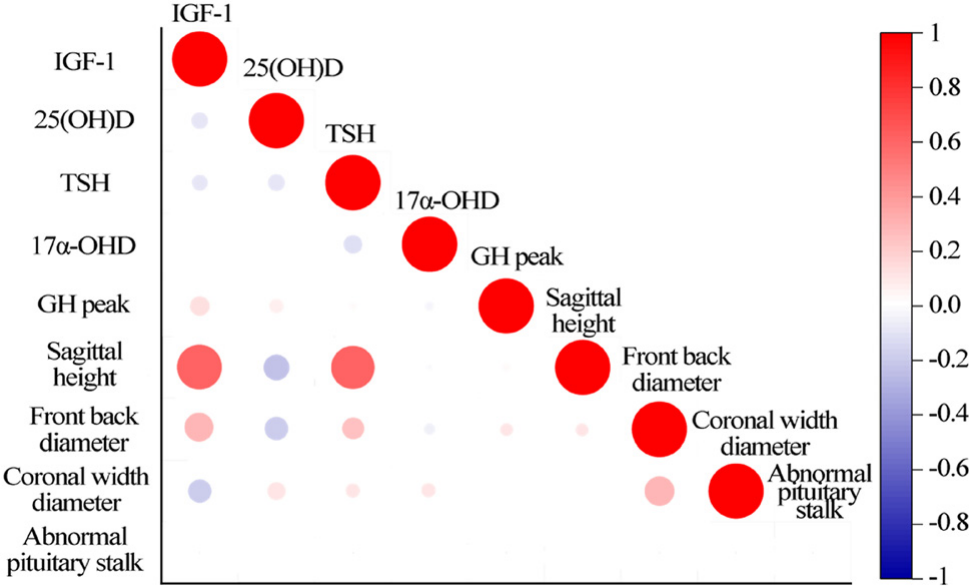
Correlation heatmap of PSH, APD, CW, PSA and short stature related serum markers.

In the correlation analysis, PSH was positively correlated with IGF-1 (r=0.381, p<0.001) and TSH (r=0.377, p<0.001), while it showed a negative correlation with 25(OH)D (r=−0.196, p=0.004). APD was positively correlated with IGF-1 (r=0.193, p=0.003) and TSH (r=0.179, p=0.044), and negatively correlated with 25(OH)D (r=−0.183, p=0.007), as shown in Figure 6.

### Relationship between pituitary volume, short stature, and related serum biomarkers

Pituitary volume was positively correlated with IGF-1 (r=0.338, p<0.001), TSH (r=0.582, p<0.001), and GH peak value (r=0.127, p=0.042), while it exhibited a negative correlation with 25(OH)D (r=−0.229, p=0.001). In the ROC curve analysis, the AUC for pituitary volume in predicting short stature was 0.647 (p<0.001), with a sensitivity of 52.04 % and specificity of 71.67 %.

## Discussion and conclusion

The hypothalamic pituitary growth axis is vital in regulating the growth of children. Defects in various factors such as GH and receptors, IGF-1 and receptors on this axis can lead to short stature. The PG is located on the ventral side of the hypothalamus and is the most complex endocrine gland in the body. Its secretion of hormones is closely related to the growth of bones and soft tissues. This study considered image quality and safety when measuring PSH, APD, and CW, and used MRI for image acquisition. On the one hand, MRI can clearly display pituitary tissue without bone artifacts, ensuring higher image quality. On the other hand, MRI examination is radiation free, which can avoid radiation damage to children and even obtain high-resolution images without using contrast agents [15].

This study divided children below the SD multiple of average height into three groups and analyzed their MRI pituitary characteristics. It was found that only PSH and pituitary volume showed significant differences among the three groups, while there was p>0.05 in the incidence of APD, CW, and PSA. Compared with the <−2SD, the PSH and pituitary volume were lower in the 2SD–3SD and >−3SD Research has found that the PSH of child with idiopathic growth hormone deficiency is lower than that of healthy children [16], and height defects are more pronounced in children with PSH <3 mm [17]. PSH and morphology change with age from infancy to adolescence. In this study, PSH was positively correlated with the age of the affected children, but the correlation was weak. The reason for this is that pituitary developmental disorders result in the PSH of children not reaching the corresponding age range, leading to a decrease in the difference in PSH between children of different age groups and a decrease in the correlation between the two parameters. A decrease in PSH can affect the secretion of growth and development related hormones. In this study, the PSH of the children was positively correlated with IGF-1 (r=0.381) and TSH (r=0.377), and negatively correlated with 25(OH)D (r=−0.196). This result confirms that a decrease in PSH hinders the secretion of growth and development related hormones, and also reflects the diversity of short stature pathways caused by a decrease in PSH. Possible reasons for the association between PSH and short stature include multiple physiological and developmental mechanisms. First, the decrease in PSH may reflect dysplasia or structural abnormalities in the pituitary gland, resulting in insufficient synthesis and secretion of growth hormone. Growth hormone is a key factor in promoting growth and bone development in children, and its insufficient secretion can directly lead to growth retardation and short stature. In addition, the decrease of PSH may also be related to the dysregulation of the hypothalamus to the pituitary gland, such as insufficient secretion of growth hormone-releasing hormone (GHRH) or excessive inhibition of somatostatin, which further affects the physiological function of GH. Finally, changes in PSH may interact with individual genetic background, nutritional status and other endocrine factors to lead to the occurrence of short stature. Therefore, the study of the relationship between PSH and short stature can provide a new perspective for clinical diagnosis and intervention of short stature.

Research suggests that pituitary volume can serve as an effective biomarker for supplementing isolated growth hormone deficiency in specific patients [18]. Unlike this study, the formula used to calculate pituitary volume in the former study was (height × width) × 3/2. The results of the two studies are roughly similar. In this study, the pituitary volume of patients in −2SD∼−3SD and >−3SD groups was significantly lower than that in <−2SD The hypothalamus plays a dual role in regulating the synthesis and secretion of GH in the anterior pituitary by secreting GHRH and somatostatin. This mechanism promotes the synthesis and secretion of growth hormone by stimulating GH cells in the anterior pituitary gland through GHRH. Meanwhile, growth inhibitory hormone maintains the balance of growth hormone in the body by inhibiting GH secretion, providing necessary endocrine regulation for promoting growth and development. Subsequently, GH enters the bloodstream and, under the mediation of GH binding proteins, binds to GH receptors in the liver, activating the Janus kinase/signaling and transcriptional activator protein (JAK-STAT) signaling pathway and stimulating the synthesis of IGF-1. GH and IGF-1 activate downstream signaling pathways together, stimulating cell proliferation and differentiation in various target tissues, thereby achieving the growth of muscle and bone related tissues in the body [19]. Shortly after birth, GH is secreted in a pulsatile manner. The GH-IGF-1 axis and nutrition jointly promote rapid early growth in infants, until the peak GH levels and IGF-1 in the body during puberty, to accelerate physical development. TSH is also secreted by the PG and participates in the metabolism of thyroid epithelial cells, promoting the synthesis of nucleic acids and proteins within cells. It is closely related to the secretion of growth hormone releasing peptides [20]. Excessive or insufficient TSH levels can lead to delayed growth and development. The pituitary volume was positively correlated with the GH peak values of IGF-1, TSH, and GH-STs in pediatric patients, consistent with previous research results. However, compared to the peak values of IGF-1 and GH, the correlation coefficient between pituitary volume and TSH was higher. This indicates that the pathogenesis of most short stature patients in this study may be related to abnormal TSH secretion caused by pituitary hypoplasia.

In addition, there has no significant discrepancy in the occurrence of APD, CW, and PSA between the <−2SD, −2SD∼−3SD, and >−3SD groups. Currently, there is limited research on the relationship between short stature, APD, and CW. Research on PSA has found that pituitary stalk interruption syndrome is closely related to short stature [21], 22]. Domestic reports indicate that most children with short stature are accompanied by pituitary stalk interruption syndrome. However, in this study, only 2 children had this symptom, and 1 was a non-short stature child with developmental delay. This differs from previous research results. To further clarify the relationship between PSH, APD, CW, PSA and short stature, this study used ROC curves to analyze the performance of predicting short stature. The AUC of predicting short stature using only sagittal PSH, pituitary volume, and four pituitary related indicators was >0.6 and <0.7. This suggests that the above indicators can be used as indicators for predicting short stature, but their predictive performance is poor, so they may become a non-specific auxiliary indicator for diagnosing short stature. Especially PSH, its sensitivity to predicting short stature was 89.29 %.

Summary: This study used MRI to measure the PSH, APD, and CW, observe PSA, and explore the relationship between the above indicators and short stature. As a result, it was found that there were significant differences in PSH and pituitary volume between <−2SD, −2SD∼−3SD, and >−3SD groups, and they were negatively correlated with short stature. Through analyzing ROC curve, both PSH and pituitary volume predicted the AUC of short stature with AUC >0.6 and <0.7. This indicates that although PSH and pituitary volume can serve as auxiliary indicators for diagnosing short stature, their predictive performance is poor. In addition, this study has many limitations. For example, after including the sample, it was found that most of the children had varying degrees of data loss, resulting in some children not being included in the study. This may lead to significant errors in the data analysis results, and the source of the included sample is too single. Therefore, prospective multi-center studies are needed in the future to avoid errors caused by missing data, to further verify the relationship between PSH, APD, CW, PSA and short stature, and provide a basis for clinical identification of the occurrence of short stature.
